# Wavelet and time-based cerebral autoregulation analysis using diffuse correlation spectroscopy on adults undergoing extracorporeal membrane oxygenation therapy

**DOI:** 10.1371/journal.pone.0299752

**Published:** 2024-10-29

**Authors:** Irfaan A. Dar, Imad R. Khan, Thomas W. Johnson, Samantha Marie Helmy, Jeronimo I. Cardona, Samantha Escobar, Olga Selioutski, Mark A. Marinescu, Chloe T. Zhang, Ashley R. Proctor, Noura AbdAllah, David R. Busch, Ross K. Maddox, Regine Choe

**Affiliations:** 1 Department of Biomedical Engineering, University of Rochester, Rochester, New York, United States of America; 2 Department of Neurology, University of Rochester Medical Center, Rochester, New York, United States of America; 3 School of Medicine and Dentistry, University of Rochester Medical Center, Rochester, New York, United States of America; 4 Clinical and Translational Sciences Program, University of Rochester, Rochester, New York, United States of America; 5 Department of Neurology, University of Mississippi, Jackson, Mississippi, United States of America; 6 Department of Medicine, University of Rochester Medical Center, Rochester, New York, United States of America; 7 Department of Biology, University of Rochester, Rochester, New York, United States of America; 8 Departments of Anesthesiology and Pain Management, Neurology and Biomedical Engineering, University of Texas Southwestern Medical Center, Dallas, Texas, United States of America; 9 Department of Neuroscience, University of Rochester Medical Center, Rochester, New York, United States of America; 10 Department of Electrical and Computer Engineering, University of Rochester, Rochester, New York, United States of America; Albert Einstein College of Medicine, UNITED STATES OF AMERICA

## Abstract

**Introduction:**

Adult patients who have suffered acute cardiac or pulmonary failure are increasingly being treated using extracorporeal membrane oxygenation (ECMO), a cardiopulmonary bypass technique. While ECMO has improved the long-term outcomes of these patients, neurological injuries can occur from underlying illness or ECMO itself. Cerebral autoregulation (CA) allows the brain to maintain steady perfusion during changes in systemic blood pressure. Dysfunctional CA is a marker of acute brain injury and can worsen neurologic damage. Monitoring CA using invasive modalities can be risky in ECMO patients due to the necessity of anticoagulation therapy. Diffuse correlation spectroscopy (DCS) measures cerebral blood flow continuously, noninvasively, at the bedside, and can monitor CA. In this study, we compare DCS-based markers of CA in veno-arterial ECMO patients with and without acute brain injury.

**Methods:**

Adults undergoing ECMO were prospectively enrolled at a single tertiary hospital and underwent DCS and arterial blood pressure monitoring during ECMO. Neurologic injuries were identified using brain computerized tomography (CT) scans obtained in all patients. CA was calculated over a twenty-minute window via wavelet coherence analysis (WCA) over 0.05 Hz to 0.1 Hz and a Pearson correlation (DCSx) between cerebral blood flow measured by DCS and mean arterial pressure.

**Results:**

Eleven ECMO patients who received CT neuroimaging were recruited. 5 (45%) patients were found to have neurologic injury. CA indices WCOH, the area under the curve of the WCA, were significantly higher for patients with neurological injuries compared to those without neurological injuries (right hemisphere p = 0.041, left hemisphere p = 0.041). %DCSx, percentage of time DCSx was above a threshold 0.4, were not significantly higher (right hemisphere p = 0.268, left hemisphere p = 0.073).

**Conclusion:**

DCS can be used to detect differences in CA for ECMO patients with neurological injuries compared to uninjured patients using WCA.

## Introduction

Veno-arterial Extracorporeal Membrane Oxygenation (VA ECMO) is a therapy of last resort for patients that have undergone acute cardiac failure. ECMO circulates blood via a pump from the patient through a gas exchange unit, removing carbon dioxide and infusing oxygen. This provides temporary mechanical support for respiratory and/or cardiac function [1–3]. While ECMO can be life-saving, up to 30% of these patients incur neurologic injuries as a result of their acute cardiogenic shock or ECMO therapy itself [4–6]. Patients with cardiogenic shock and cardiac arrest often experience disrupted cerebral autoregulation (CA), a physiologic mechanism that constricts and dilates cerebral vasculature to stabilize cerebral blood flow (CBF) over a range of mean arterial pressure (MAP) [7]. Impaired CA leads to direct correlations between MAP and CBF. Impaired CA is a marker of acute brain injury and can worsen the existing neurological damage [8–11]. Measuring CA in acutely brain-injured patients can allow clinicians to ascertain the MAP range that optimizes CBF at any given time, guiding precision-based ECMO care [12,13]. This is especially important in ECMO patients as clinicians control systemic perfusion via the circuit pump speed in addition to vasopressors.

CA measurement has most often been described using invasive measurement of intracranial pressure (ICP), which is risky in ECMO patients because ECMO-induced coagulopathy raises the risk of intracranial hemorrhage [14,15]. Near-infrared spectroscopy (NIRS) has been increasingly used to measure CA in these patients non-invasively, but commercially available NIRS can be inaccurate [16–18] since it uses blood oxygenation as a surrogate to CBF instead of measuring CBF directly.

Diffuse correlation spectroscopy (DCS) is a non-invasive optical monitoring tool that measures CBF by detecting the scattering of light off mobile red blood cells in the brain tissue. This produces a blood flow index (BFI) proportional to the blood flow in tissue [19]. This has been validated against gold-standard CBF monitoring techniques including xenon-CT and arterial spin-labeled MRI [20,21]. Furthermore, DCS has been used to monitor CBF in neonates and adults with neurological injuries, and measure CA in non-ECMO adults and neonates undergoing ECMO [22–27]. DCS also allows measurement of perfusion in the microvasculature of the cerebral cortex which is a site of injury of anoxic brain injury (ABI) that is prevalent in ECMO patients, unlike TCD which measures perfusion from the large cerebral arteries. Studies have demonstrated microvascular perfusion deficits in patients with ABI [28].

CA is most often measured using a moving Pearson correlation (or index) over a fixed time window (e.g., five minutes) between MAP and a brain-specific parameter, such as ICP (yielding a pressure reactivity index) or CBF with Transcranial Doppler (TCD) or NIRS [10,29–31]. A low index value denotes intact CA, while a high index value indicates impaired CA. Pearson correlation between MAP and DCS-derived CBF is denoted as DCSx.

To reduce the influence of noise, DCSx is only quantified when MAP changes are significant [32,33]. ECMO patients often experience minimal changes in MAP as their blood pressures can be largely dependent on a pump rate set by the clinician. Thus, there are large periods of time where DCSx is not calculated as MAP does not change. To address this issue, we quantified CA using wavelet coherence analysis (WCA) which uses a wavelet transform to measure the coherence in the frequency domain between relative BFI (rBF) and MAP [34]. Unlike DCSx, WCA does not require a fixed threshold for MAP change since it uses surrogate data for rigorous testing of significant coherence (i.e., whether the coherence is due to the signal and not due to noise). In addition, WCA provides coherence values between MAP and rBF signals at different frequencies, which is unavailable in DCSx. WCA has previously been used to measure CA using NIRS-derived cerebral oxygen saturation and MAP, finding increased coherence in patients with brain injuries [35–38]. While promising, these studies used brain tissue oxygenation to infer CBF, rather than measuring CBF directly using DCS.

In this study, we compared two methods of measuring cerebral dysregulation, DCSx and WCA, in adult ECMO patients with and without acute brain injury using DCS to directly measure CBF.

## Methods

### Ethics statement

The study was conducted according to a protocol approved by the University Research Studies Review Board (RSRB) at the University of Rochester (Permit number: RSRB00003324). Recruitment started on 12/01/2019 and ended on 6/30/2023. Written consent was obtained for each subject.

### Eligibility and recruitment

Eligible patients were adults ≥ 18 years old who were undergoing VA ECMO treatment. Patients were excluded from the study if they had a preexisting neurological injury at the start of ECMO initiation, or skin injuries that impeded DCS measurements. Of the 21 total patients that were recruited, 11 patients received CT imaging during their hospitalization and were selected for this study.

### Hardware setup and data recording

The DCS instrument is based on a design detailed in previous papers [39–41]. The instrument contains a 785 nm long coherence laser (DL785-120-SO, CrystaLaser, Reno Nevada) with two 4-channel single photon counting modules (SPCM-AQ4C, Excelitas, Waltham, Massachusetts), and normalized intensity autocorrelation curves were calculated using an 8-channel hardware correlator (Flex05OEM, Correlator.com, Bridgewater, New Jersey). Optical fibers connected to the laser source and detectors were positioned 2.5 cm apart in a rubber probe. Two probes were secured to the forehead on the left and right hemispheres using two-sided tape (#1522 and #9917, 3M, Minnesota) and Tegaderm (#16002, 3M, Minnesota) to ensure good contact with the skin. Laser power was adjusted to adhere to the American National Safety Institute (ANSI) standards [42]. With a 2 second integration time, and laser switching to each probe source, data acquisition for each hemisphere was 0.25 Hz.

BFI data, which is proportional to blood flow in the tissue was calculated from the intensity autocorrelation data acquired by the DCS device using a semi-infinite homogenous model with an assumed absorption coefficient of 0.1 cm^-1^ and reduced scattering coefficient of 10.0 cm^-1^. Relative blood flow (rBF) was calculated using the median value as a baseline as described in our previous papers [39,40].

Continuous MAP data was recorded from Philips Intellivue MX800 monitors (Philips Healthcare, Andover, MA) using Medicollector software (Medicollector, Winchester, MA). The data from the monitor was acquired at 1 Hz and was time-synchronized with rBF data. Afterwards, MAP data was downsampled to match the rBF data sampling rate of 0.25 Hz. Demographic data was obtained from a medical chart review, and CT scan results were documented from the radiologist’s findings.

### DCSx calculation

**Fig 1:**
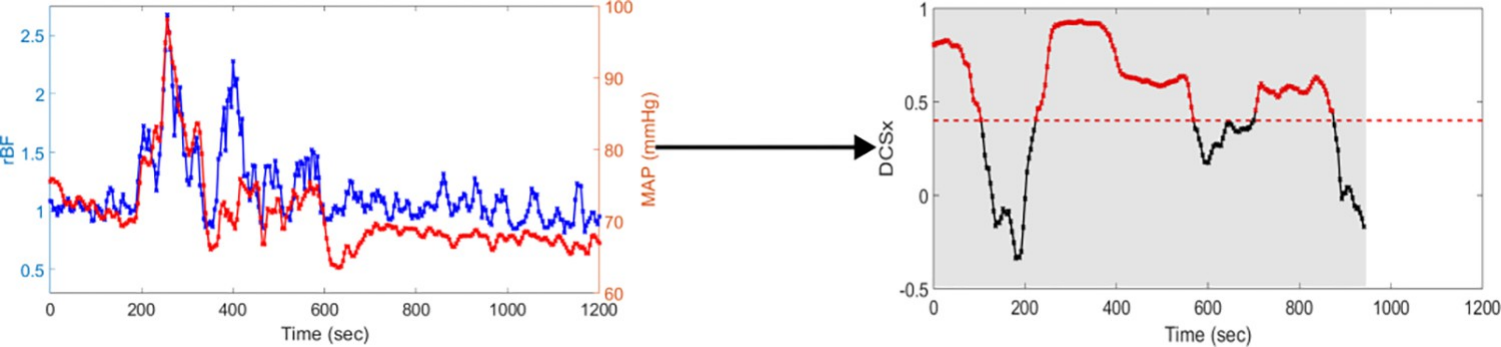
Example of DCSx. The time series on the left is used to calculate the DCSx time series on the right. DCSx is calculated only during segments when the mean arterial pressure had a range greater than 5 mmHg. The dashed red line indicates threshold set at 0.4 and the red dots indicate DCSx values above this threshold. %DCSx, the percentage of DCSx values above 0.4 of the total time DCSx is calculated, 66.5% for this segment.

To derive the autoregulation index DCSx, a moving Pearson correlation coefficient over a five-minute window is calculated as described in a previous paper [39]. To reduce the impact of the noise in the signal, the correlation coefficient is only taken when the MAP has a range of at least 5 mmHg in the five-minute window. An example of DCSx calculation is shown in Fig 1. Cerebral dysregulation was defined as DCSx above a threshold value of 0.4 [29,30,43]. The percentage of time DCSx is above this threshold value, defined as %DCSx, is then determined for each twenty-minute time segment when DCSx exists. Data segments that are less than twenty minutes are discarded. As shown in Fig 1, DCSx is calculated for the first 1000 seconds of the signal shown in the shaded region due to the MAP range in the window exceeding the 5 mmHg threshold. However, for the last 200 seconds, DCSx is not calculated since the MAP range does not meet the 5 mmHg threshold even though there are noticeable changes in rBF. The %DCSx calculated for the time segment shown in Fig 1 was 66.5%.

### Wavelet coherence calculation

**Fig 2:**
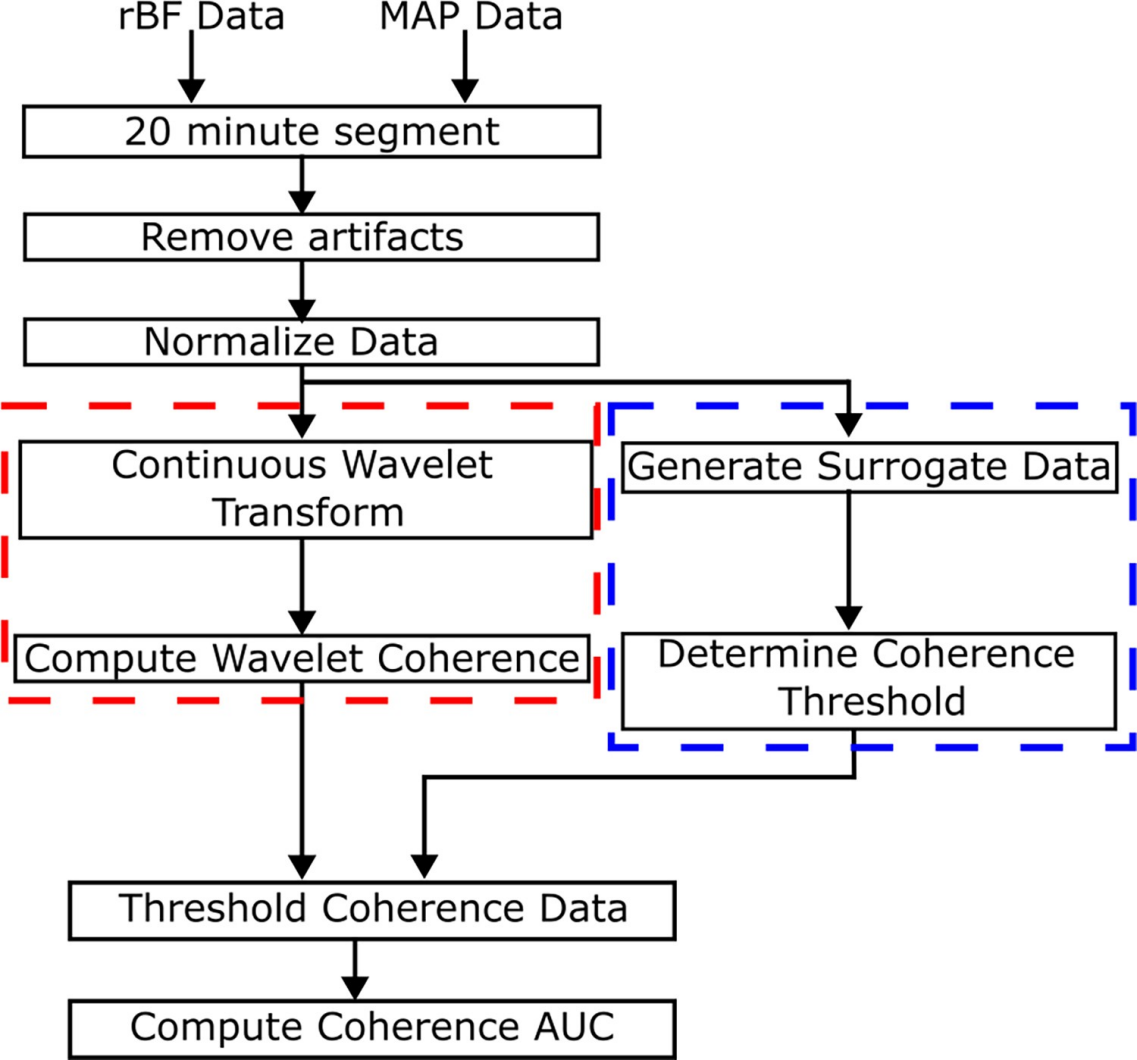
The process to determine the wavelet coherence. First the data is segmented into 20-minute sections, and then artifacts are identified and removed before normalization. The continuous wavelet transform is performed, and the coherence is then calculated (Red box). To determine if the coherence is significant, coherence data is generated from surrogate data (Blue box). Using the surrogate data, a threshold is applied to the coherence data and the area under the curve (AUC) is then calculated. (For details of surrogate data, see Fig 4).

The WCA process is shown in Fig 2. First, both daily measurements of rBF and MAP were divided into multiple segments lasting twenty minutes each. Data segments that are less than twenty minutes are discarded. Data points manually identified as motion artifacts were discarded from the analysis. Each segment was then normalized to have a mean of zero and a standard deviation of one. The continuous wavelet transform for the rBF and MAP data was computed using the *cwt* function in MATLAB using the complex Morse mother wavelet [34]. Using Eq 1, the coherence R^2^(*f)* is calculated:

$$R^{2}(f)=\frac{\left|E\langle W_{rBF}^{*}(f,t)W_{MAP}(f,t)\rangle \right|}{\sqrt{E\langle W_{rBF}^{*}(f,t)W_{rBF}(f,t)\rangle E\langle W_{MAP}^{*}(f,t)W_{MAP}(f,t)\rangle }}$$ (1)

where *W(f*,*t)* is the continuous wavelet transform of either the rBF or MAP data. E<> is the average across time and R^2^(*f*) is the coherence between the two signals at each frequency *f*. R^2^(*f*) values range from 0 to 1, where 0 is no coherence and 1 is perfect coherence.

**Fig 3:**
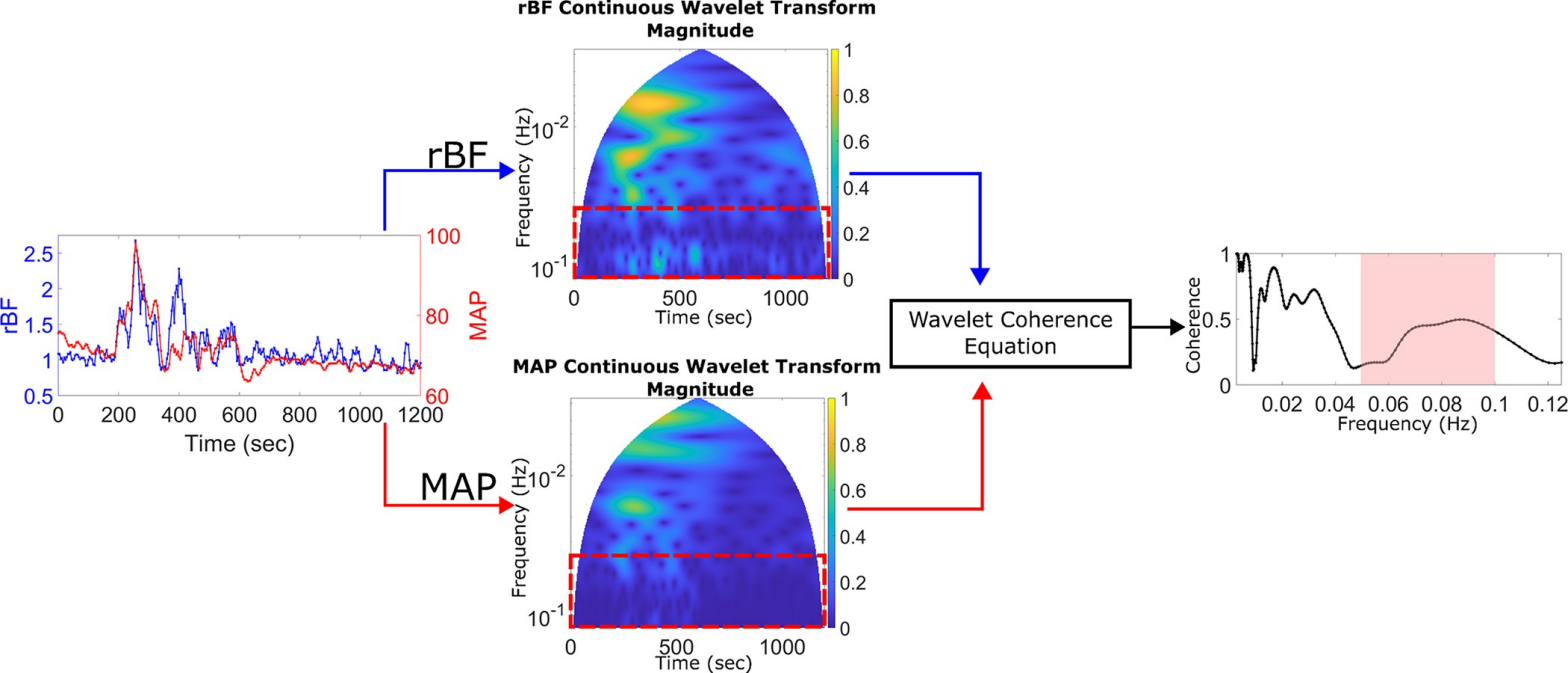
An example of how the wavelet coherence is calculated. First the continuous wavelet transform (middle) of the rBF and MAP time series (left) is calculated. Using the wavelet coherence equation and the continuous wavelet transform, the coherence vs frequency is calculated (right). The red boxes in the middle figures and red shade in the right figure denote the 0.05 to 0.1 Hz range that is used in the analysis.

Fig 3 shows an example of how the coherence is calculated. The segmented signals of rBF and MAP are projected into the time-frequency space using a continuous wavelet transform. By looking at the magnitude of the wavelet transform, one can see the contributions of each frequency at a specific time. The wavelet transform data is then input into Eq (1) to compute the coherence, and the coherence values are determined at each frequency as shown in Fig 3. To ensure that the numerator and denominator do not cancel each other out, a smoothing operation is included in the coherence calculation, which takes the expected value, E<> across all time. Our analysis focuses on the frequency scale from 0.05 Hz to 0.1 Hz shown in Fig 3 as the red dotted line. Frequencies lower than 0.05 Hz are discarded due to the length of the time series length being too short to evaluate those frequencies. By dividing the data into twenty-minute segments, at least ten nonoverlapping cycles of the wavelet in the time domain for each frequency are present, allowing for multiple independent calculations of the coherence. Adjusting the width of the Morse wavelet to meet this constraint is achieved by setting the *TimeBandwidth (TBW)* parameter for the *cwt* MATLAB function. The width of the wavelet in the time domain is equal to $2(f_{c}/f)\sqrt{(TBW)/2}$, where *f_c_* is the center frequency of the wavelet and *f* is the desired frequency. Furthermore, 0.05 Hz corresponds to the frequency limit used in previous wavelet coherence studies that used near-infrared spectroscopy [44–46]. The upper limit of 0.1 Hz is based on the wavelet frequency spectrum. To ensure that the wavelet’s frequency response bandwidth does not exceed the Nyquist limit (0.125 Hz for this study), MATLAB limits the upper resolvable center frequency of the wavelet such that the magnitude at the Nyquist limit is 50% of the peak magnitude at the center frequency. The center frequency at which this occurs at is 0.1 Hz for this study. Wavelets with a center frequency higher than 0.1 Hz will have a larger portion of their frequency spectrum past the Nyquist limit, which causes aliasing and influences the coherence results.

**Fig 4:**
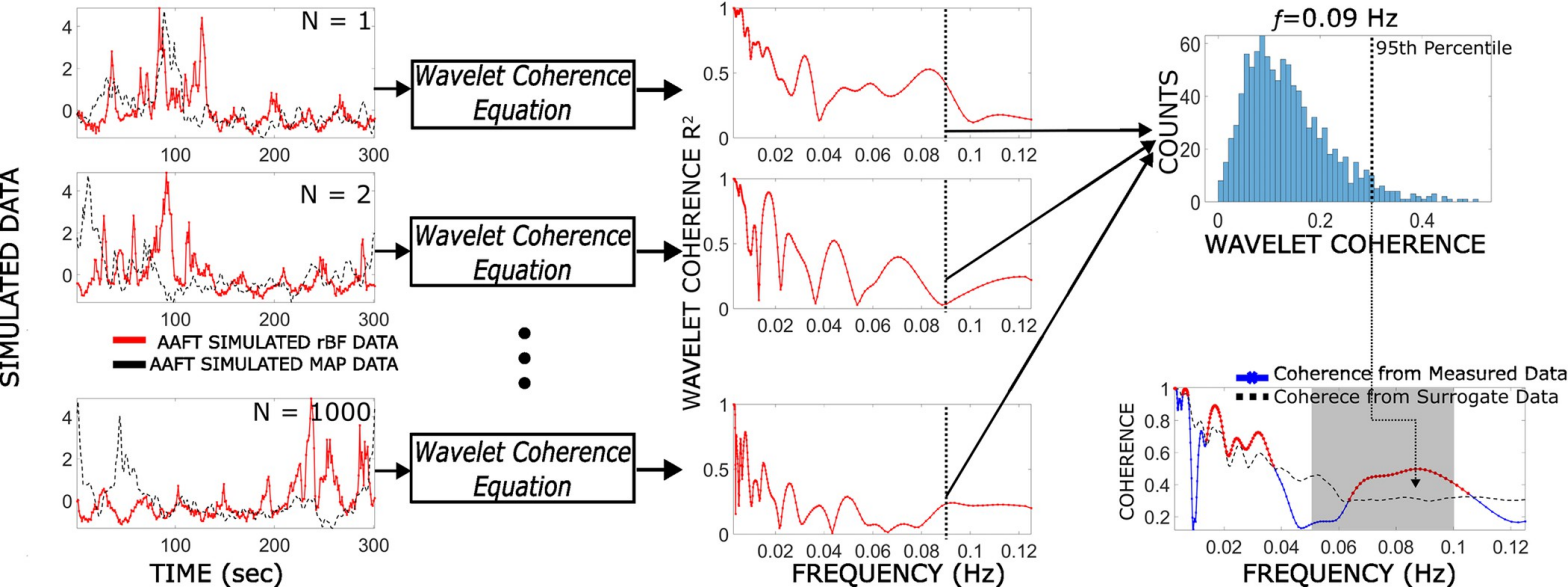
An example of how the surrogate data is used for determining whether coherence at each frequency is due to noise or not. First the time series for both rBF and MAP are randomized using the iterative amplitude adjusted Fourier transform (left). Using these time series and the wavelet coherence equation (Eq 1), the coherence is calculated for the surrogate data (middle). This is done 1000 times to generate a distribution. Using signals at 0.09 Hz as an example, the right uppermost figure shows the distribution of coherence values and determination of the 95^th^ percentile of coherence at that frequency. The actual coherence is then compared to the 95^th^ percentile of surrogate at each frequency and if greater, it is considered significant (bottom right). Values that are above the threshold are determined as significant coherence values and are highlighted as the red bolded line and those in the shaded region are used for the WCOH calculation.

To evaluate if the coherence is due to actual fluctuations in the signal and not due to noise, surrogate testing is done using iterative amplitude adjusted Fourier transform (iAAFT). One thousand surrogate signals for both MAP and rBF are generated by randomizing the phase of the original data signal thus generating a new signal with the same Fourier spectra as the original signal shown on the left side of Fig 4. Using this surrogate data and the same methodology, a distribution of wavelet coherence values is generated at each frequency as shown in the upper right corner of Fig 4. From the distribution, the value at the 95^th^ percentile is determined and used as a threshold value. If the coherence from the original signals is above these values at each frequency, then the coherence is determined to be significant and due to real changes in the signals. Then, the area under the curve of the significant wavelet coherence, defined as WCOH, is calculated by taking the integration using the numerical trapezoidal method between 0.05 to 0.1 Hz only for frequencies where coherence is higher than the 95^th^ percentile from the surrogate distribution [47].

### Neurological injury assessment

Injuries defined as anoxic brain injury (determined based on loss of gray-white differentiation, loss of sulcal definition, and cerebral edema), hemorrhage, infarction, herniation, or hydrocephalus were noted on the radiological diagnostic reports based on the CT scan. CT scans were obtained as part of routine clinical care prior to ECMO initiation or if clinically indicated due to change in neurologic exam. Board-certified neuroradiologists reviewed CT scans at the hospital.

### Statistical analysis

The mean values for the WCOH and %DCSx analyses were calculated from all segments for the entire monitoring period for each patient. Patients are divided into two groups based on the neurological findings of their CT imaging scans. One group consisted of patients with neurological injuries identified on CT imaging, while the other group consisted of patients without any radiographic injuries. Our primary hypothesis was that ECMO patients with neurological injuries have higher WCOH and %DCSx for both hemispheres than those without neurological injuries. Statistical testing was then performed using a one-sided non-parametric Wilcoxon rank-sum test after testing for normality. P-values less than 0.05 are determined to be significant. A two-tailed Student’s t-test was used to test for differences in demographic data.

A simple logistic regression [48] was performed for the parameters separately to analyze how %DCSx compared with WCOH in identifying which patients had a neurological injury. The logistic equation used for the fitting was defined as

$$p(x_{i})=\frac{1}{1+e^{-(\beta_{0}+\beta_{1}x_{i})}}$$ (2)

where x_i_ is the parameter value for each patient and p(x_i_) is 0 if the patient had no defined injury and 1 if the patient had a neurological injury. The parameter values are first normalized, so their distribution has a mean value of zero and standard deviation of one. The model is then fitted to determine the parameters *β*_0_ and *β*_1_ of the logistic equation. Using different thresholds for the value of p(x_i_), the output of the logistic regression is compared to measured data and the true positive rate (TPR) is plotted against the false positive rate (FPR) to provide the receiver operating characteristic (ROC) curve. The area under the curve (AUC) is then calculated to determine how well the logistic regression model can differentiate between patients with neurological injury versus those without based on WCOH or %DCSx parameters. The area is taken to determine how effective the model is at predicting if a patient has a neurological injury based on the results from WCOH or %DCSx analysis. For each WCOH and DCSx ROC curves based on the logistic equation, the optimal threshold value to differentiate uninjured and neuroinjured patients was determined by finding the maximum Youden’s J index corresponding to the farthest distance from the ROC line to the asymptote [49].

## Results

Eleven patients who underwent VA ECMO were analyzed in this study. Six patients were determined to have no neurological injuries based on CT results while the other five patients had indication of a neurological injury. Table 1 displays the demographic characteristics for each patient. The mean age ± standard deviation was 55.0 years ± 17.1 years. Statistical testing using a two-tailed t-test showed no difference in age between groups. Eight patients were admitted to the hospital for cardiac arrest, and three were admitted for cardiogenic shock. Of the eleven patients, 9 (82%) were male and 2 (18%) were female. Brain CT images showed that four patients suffered from ABI and one patient had a microhemorrhage.

**Table 1:** Demographic data of the eleven patients under VA ECMO monitored for this study.

Subject #	Age	Sex	Indication for ECMO	Radiographic Brain Injury
1	30	female	cardiogenic shock	None
2	73	male	cardiac arrest	None
3	64	male	cardiogenic shock	None
4	65	male	cardiac arrest	None
5	79	male	cardiogenic shock	None
6	48	male	cardiac arrest	None
7	53	male	cardiac arrest	ABI (bilateral)
8	21	female	cardiac arrest	ABI (bilateral)
9	58	male	cardiac arrest	ABI/Edema (bilateral)
10	57	male	cardiac arrest	ABI (bilateral)
11	57	male	cardiac arrest	Microhemorrhage (Right)

Patients with neurological injuries are shaded and show what injury was identified on the computed tomography (CT) scan. Additionally, the reason for admission to the hospital and indication for ECMO is described. ABI: Anoxic brain injury.

Table 2 shows the timing of DCS and CT with respect to ECMO, along with Glascow Coma Scale (GCS) at discharge. On average, DCS monitoring started 2 days after ECMO initiation, for two hours daily for five days during ECMO therapy. ECMO settings were mostly static during each two-hour DCS monitoring session. CT scans were obtained during ECMO for each patient, on average 3 days after ECMO initiation. Of the six patients without radiographic brain injury, 50% of them had CT taken during the DCS monitoring period. Subject 3 had CT taken 1 day after DCS. For subjects 2 and 6, multiple CT findings before and after DCS confirmed the absence of brain injury. Of the five patients with radiographic brain injury, CT imaging was performed during the DCS monitoring period for four patients. For subject 10, CT images taken 1 day before and 1 day after DCS monitoring period both showed ABI. Neurologic outcomes, based on GCS after decannulation of ECMO or on the last day of ECMO for those who could not be decannulated (denoted as final GCS), did not always align with CT-detected brain injury status during ECMO. The final GCS for subject 4 (part of the uninjured group) was low due to sepsis causing severe encephalopathy. Subjects 7 and 11 (part of the neuroinjured group) had poor GCS at the ECMO start (not shown) but their GCS improved throughout the ECMO session, and they had awakened by the time the final GCS was assessed.

**Table 2:** Measurement timing and neurologic assessment with GCS.

Subject #	DCS: days after ECMO start	DCS period (days)	CT: days after ECMO start	CT with respect to DCS	Decannulated?	Final GCS
1	2	5	4	Day 3 of DCS	Yes	14 (6)
2	2	3	0 14	2 days before, 11 days after	Yes	11 (6)
3	4	4	8	1 day after	No	13 (6)
4	2	4	4	Day 3 of DCS	No	3(1)
5	2	6	4	Day 3 of DCS	Yes	11 (6)
6	1	5	0 21	1 day before, 15 days after	Yes	6 (4)
7	3	8	1	Day 1 of DCS	Yes	11(6)
8	0	7	2	Day 3 of DCS	Yes	5(2)
9	2	5	5	Day 4 of DCS	No	3(1)
10	1	2	0 3	1 day before, 1 day after	No	3(1)
11	0	5	0	Day 1 of DCS	No	11 (6)

Subjects with neurological injuries are shaded. Timing information of CTs performed closest to DCS monitoring are presented. For GCS, total score is shown along with motor score in parentheses. Motor score is presented in case GCS verbal score is not available due to intubation. For those who were decannulated from ECMO, the best GCS after decannulation is reported as final GCS. Those who were not decannulated, GCS on the last day of ECMO therapy is reported as final GCS.

Table 3 displays the percentage of total monitoring time used to calculate each parameter. The second column shows how much time the MAP range was greater than 5 mmHg and thus used to calculate DCSx versus the total time. The third column details how much time wavelet coherence was calculated for each subject. Note that artifact removal resulted in some wavelet coherence data being excluded and thus the % of total time does not always equal 100%. Overall, more time was used to compute the wavelet coherence when compared to DCSx.

**Table 3:** Percentage of total time each parameter was calculated for each subject.

Subject #	% of total time selected for DCSx calculation (MAP > 5mmHg)	% of total time selected for wavelet coherence calculation
1	33.0%	100.0%
2	36.8%	98.6%
3	41.2%	98.3%
4	34.9%	97.9%
5	95.0%	99.3%
6	39.1%	99.7%
7	69.4%	99.1%
8	54.3%	99.4%
9	26.0%	99.6%
10	65.3%	99.2%
11	35.1%	98.9%

Subjects with neurological injuries are shaded.

**Fig 5:**
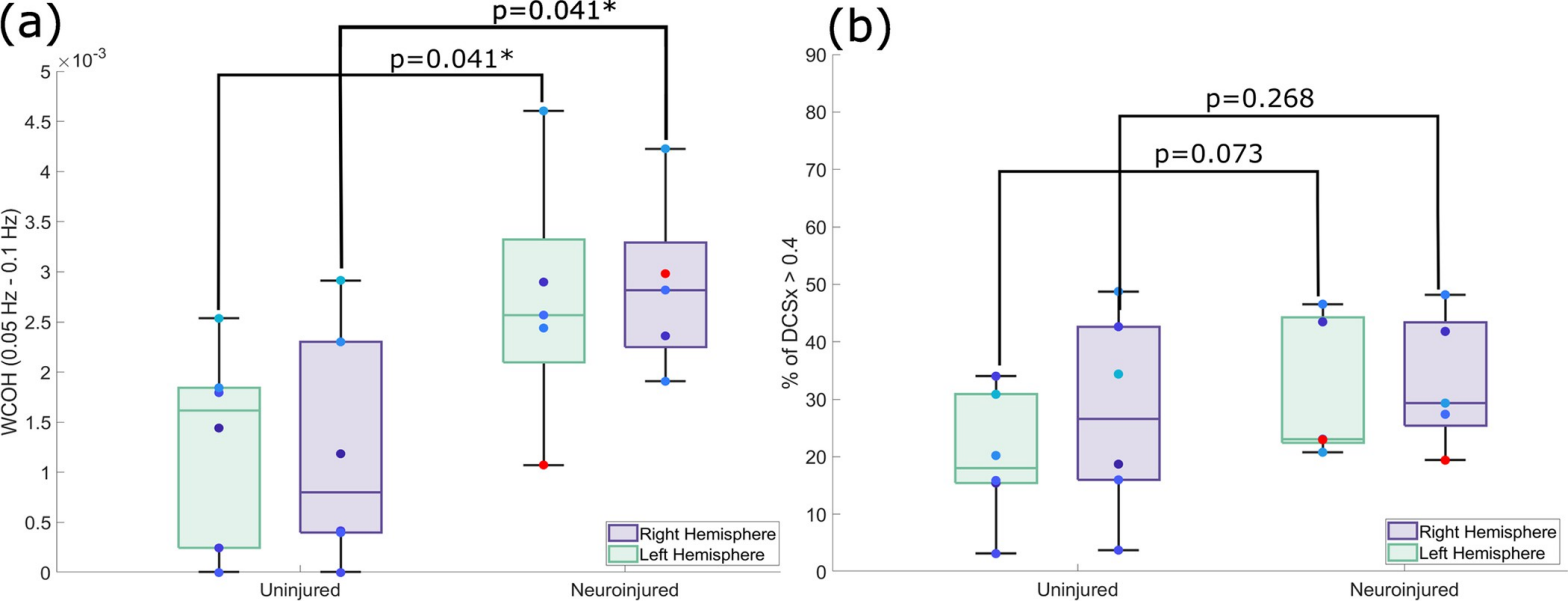
Boxplots comparing DCS-derived CA measures between neuroinjured and uninjured patients determined by CT. (a) Boxplots comparing WCOH. Neuroinjured group had a significantly higher coherence compared to uninjured for both the left (p-value = 0.041) and right (p-value = 0.041) hemispheres. (b) Boxplots comparing the %DCSx. Neuroinjured group did not show a significantly higher %DCSx compared to uninjured for the left (p-value = 0.073) and right (p-value = 0.268) hemispheres. Significance was computed using a one-tailed Wilcoxon rank-sum test. Red data point indicates subject 11 who was diagnosed with a microhemorrhage in the right hemisphere.

Fig 5A displays the WCOH value between 0.05 to 0.1 Hz for the patient group with normal CT scans and the patient group with neurological injuries identified by CT scans. The WCOH values were significantly higher for the ECMO patients with neurological injuries identified by CT for both the left (p = 0.041) and right (p = 0.041) hemispheres than those with no injuries. Fig 5B displays the %DCSx for both groups. No significant difference in %DCSx was found between the injured and uninjured group in either hemisphere.

Note that subject 11’s WCOH value for the right hemisphere (0.003) is higher than the median value for the uninjured group, while their WCOH value for the left hemisphere (0.001) is similar to the median value of the uninjured group, as shown as the red dots in Fig 5. If the left hemisphere value of subject 11 is categorized to the uninjured group since there was no microhemorrhage in the left hemisphere, Wilcoxon rank-sum test for WCOH and %DCSx of left hemisphere improves to p = 0.005 and p = 0.051 respectively.

**Fig 6:**
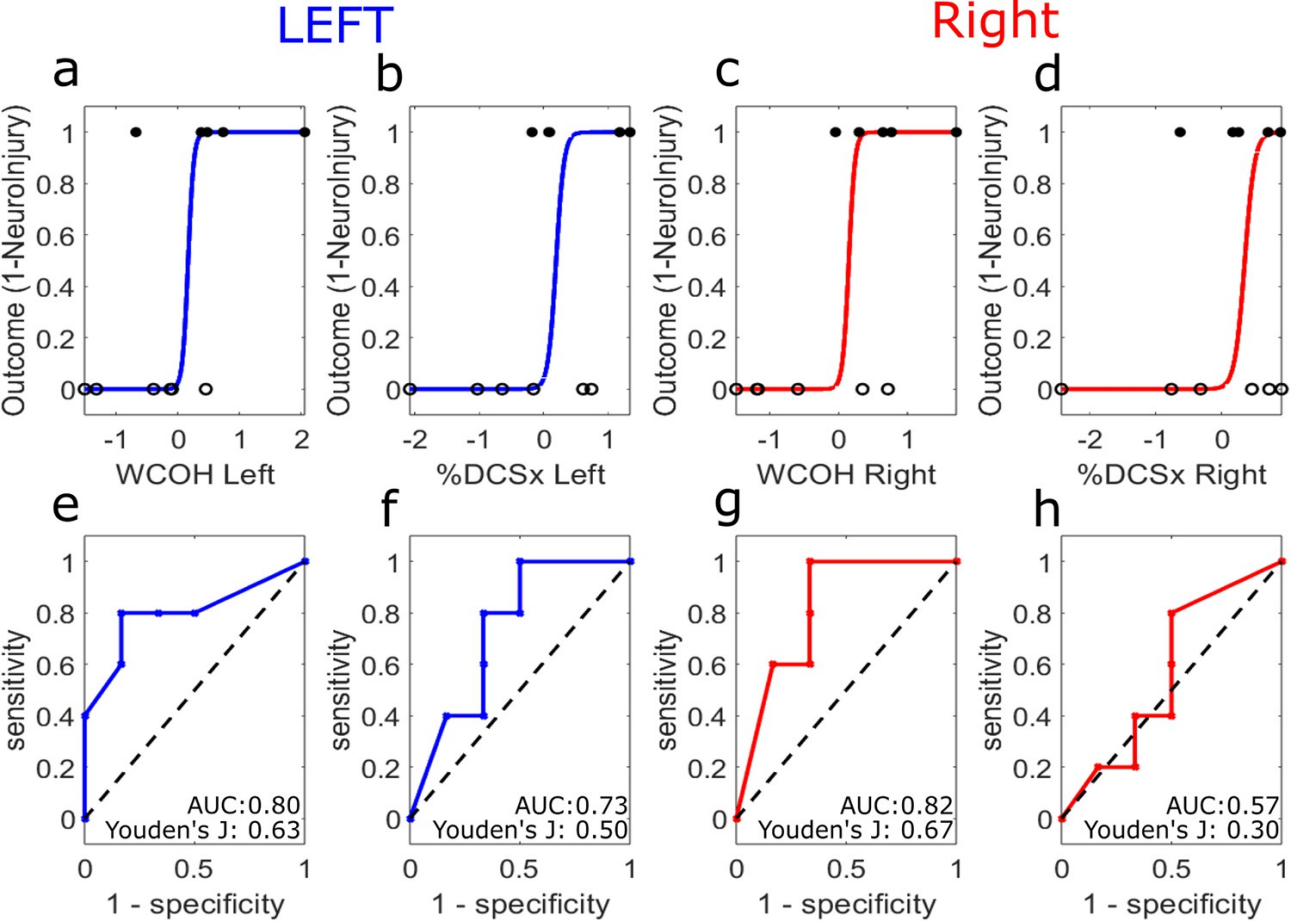
Logistic regression and ROC curves. Logistic regression for (a) WCOH left hemisphere, (b) %DCSx left hemisphere, (c) WCOH right hemisphere, and (d) %DCSx right hemisphere are presented in the top row. Hollow black circles at 0 represent values for patients with no neurological injuries, while filled circles at 1 represent values for patients with neurological injuries. ROC curves for (e) WCOH left hemisphere, (f) %DCSx left hemisphere, (g) WCOH right hemisphere, and (h) %DCSx right hemisphere are shown in the bottom row. ROC plots false positive rate (1-specificity) against the true positive rate (sensitivity). Each parameter’s area under the curve (AUC) and maximum Youden’s J index values are reported.

Fig 6 displays the logistic regression curves for the right hemisphere’s %DCSx and WCOH predictor variables. The generated curve demonstrates how well the regression fits the data, which is displayed as the black circles at 0 and 1 on the y-axis. Circles at the 1 value represent the WCOH or %DCSx values for patients with a neurological injury, while circles at the 0 value represent WCOH or %DCSx for patients with no identifiable neurological injury. The closer the model is to the asymptote (black-dashed line), a ROC curve with an AUC of 0.5, the worse the model is at predicting outcomes based on the parameter. Thus models with a higher AUC correspond to a better estimation of neurological injury. Fig 6 displays the AUC values for all parameters (*WCOH Left AUC*: 0.80, *WCOH Right AUC*: 0.82, *%DCSx Left AUC*: 0.73, *%DCSx Right AUC*: 0.57). The WCOH AUC values were around 0.80, showing excellent discrimination between the two groups, and were consistently higher compared to the %DCSx AUC values. The maximum Youden’s J value is also displayed for all parameters in Fig 6 (*WCOH Left Youden’s J*: 0.63 at a threshold of 0.0019, *WCOH Right Youden’s J*: 0.67 at a threshold of 0.002, *%DCSx Left Youden’s J*: 0.50 at a threshold of 22.1%, *%DCSx Right Youden’s J*: 0.30 at a threshold of 23.9%). The maximum Youden’s J values for the WCOH parameters were closer to 1 (i.e., perfect classification) compared to %DCSx, thus were better able to distinguish between the two groups.

## Discussion

In this paper, we have demonstrated the differences in cerebral autoregulation for ECMO patients with neurological injuries detected by CT versus ECMO patients without, using both WCOH and %DCSx. ECMO patients with neurological injuries had a significantly higher WCOH value than those without injuries, whereas %DCSx data showed no significant difference for either hemisphere. Additionally, when looking at the logisitic regression for each parameter, WCOH showed higher AUC and maximum Youden’s J values than %DCSx, indicating that WCOH had better potential at discriminating which patients have neurological injuries.

The stastistical significance of the WCOH parameters compared to the DCSx parameters can be attributed to the underlying process of calculating wavelet coherence. Wavelet analysis provides a decomposition of the contributions of frequencies for nonlinear signals, information lost in a simple correlation between MAP and rBF employed by DCSx. Since the non-linearity and non-stationarity of the rBF and MAP signals violate the preconditions for using a Fourier transform analysis, WCA is employed [50,51]. The coherence between two signals can be established for a wide range of frequencies, and multiple studies have shown how coherence analysis relates to neurological function [5,6,35,38,44,52–54]. Low-frequency oscillations ranging from 0.05 to 0.1 Hz have been linked to specific physiological mechanisms involving the regulation of vascular smooth muscle tone in arterioles and capillary beds [35,44,45,54,55]. These studies showed that by inhibiting these mechanisms via vasodilators for vascular smooth muscles, the gain in the 0.05 to 0.1 Hz frequency spectrum would decrease. Changes in vascular smooth muscle tone via intrinsic myogenic activity serve to regulate the amount of blood flowing in the microvasculature [54]. Neuronal, astroglial, and endothelial injury may impair vasomotor tone, leading to cerebral dysregulation marked by direct correlation between systemic blood pressure and CBF [56,57]. This concept has been demonstrated in neonates and piglets where dysregulation indices were higher in brain-injured subjects compared to healthy controls [38,58]. Brain-injured subjects displayed temporal and frequency changes in MAP that drove similar time and frequency changes in CBF. Our results recapitulate this finding in humans using wavelet coherence.

In addition to the lack of frequency information, the reason that DCSx did not achieve statistical significance could be the choice of MAP threshold. In this study we used a MAP threshold of 5 mmHg to ensure that fluctuations were not due to noise. However some studies either do not use a MAP threshold [8,31] or use a more stringent one [43,59], which influences the results. As shown in Table 3, meeting the 5 mmHg threshold was difficult for ECMO patients where variation in MAP is minimal due to depressed myocardial contractility. However, DCSx still provides a quicker measure of cerebral dysregulation that can be determined in real time more easily than WCA, and could be optimized for specific applications.

The study had several limitations. The sample size for this study was small, however the preliminary data shows promising results, and the recruitment of more patients is ongoing. Regarding the patient population, the study included patients with different etiologies (cardiogenic shock vs cardiac arrest) to provide a reasonable sample size per group. In the future, limiting inclusion to single underlying etiologies for ECMO, such as cardiac arrest, may further reduce heterogeneity in pathomechanisms that cause cerebral dysfunction. One source of bias could be the limited DCS monitoring length (two hours per day) since it may result in missing time periods with large blood pressure changes. This probably affected the performance of %DCSx more than WCOH. Furthermore, not monitoring continuously may have resulted in missing acute secondary injury that could drastically increase WCOH or %DCSx. Another source of bias is the reliance on the CT imaging as the gold standard measure of brain injury. CT may not be sensitive enough to detect early manifestation of injury or certain types of brain injury [60]. Unfortunately, ECMO circuits are not compatible with magnetic resonance imaging, which has better sensitivity than CT. On the other hand, some patients with CT-detected brain injury (subjects 7 and 11) recovered the brain function during ECMO treatment and exhibited high GCS scores after decannulation or on the last day of ECMO. When WCOH values of patients with good (GCS motor score > 3) vs poor (GCS motor score ≤ 3) neurologic outcomes based on the final GCS were compared, no statistical significance was found. This may be due to using a WCOH value per patient averaged over the whole DCS monitoring period instead of analyzing its temporal evolution. In the future, we will explore if the time evolution of WCOH may reflect functional recovery with continuous monitoring for such patients.

From the technical side, we only analyzed amplitude coherence in this study, but some studies have also evaluated the phase information using a similar wavelet methodology [50,58] when searching for neurological injuries. The phase coherence analysis provides additional information about the phase relationship between the signals, and results from these papers have shown comparable findings when analyzing phase coherence and amplitude coherence for neurological injuries [50,58]. In the future we will analyze the phase information of rBF and MAP. Due to the experimental setup and hardware, the data sampling rate was limited to 0.25 Hz, which only allows WCA up to 0.1 Hz. For this study, the sampling rate was fast enough to provide analysis of the frequencies between 0.05 Hz and 0.1 Hz which is relevant to evaluate the myogenic component of CA. Future hardware upgrades for faster acquisition will enable the analysis of faster frequencies and their influences on CA. To expand the lower frequency limit, longer measurement recordings to support data segments longer than 20 minutes will be done to meet the minium number of independent wavelet cycles. Another limitation is the use of the simplified one-layer solution to calculate BFI. The method used to determine BFI in this study does not account for the influence of the skin blood flow. In the future, a multi-layer analysis will be used to remove extracerebral blood flow from the BFI calculation [61]. A limitation of the logistic regression model is the simplicity of the model. Because the model only looked at how one parameter affected the brain injury status, other patient characteristics were not considered, such as indication for ECMO or age. In the future when more patients are included, these characteristics will be considered to determine their influence on brain injury status and neurological outcome.

Despite all the limitations, using WCOH to detect neurological injuries is attractive since it can be assessed at the bedside continuously instead of intermittent snapshots of brain health by CT. Although some cardiovascular parameters could be used to predict successful weaning from VA ECMO [62,63], current clinical practice still does not take brain perfusion into consideration, leaving the brain at risk of secondary injury. Continuous neuromonitoring enables live feedback during weaning procedures, which could lead to individualized weaning protocols using cerebral perfusion targeted blood flow rates and gas exchange rates. Furthermore, our platform may improve tracking therapeutic efficacy and patient selection of current and future cerebral protective treatments (e.g., modulating carbon dioxide or oxygen, blood pressure, body temperature) for cardiac arrest patients [64–67].

## Conclusions

We have evaluated cerebral autoregulation using both DCSx based on moving Pearson correlation and WCA in adult ECMO patients with and without brain injuries defined by CT. Both methods showed higher values for brain-injured patients, indicating impaired cerebral autoregulation. WCOH outperformed %DCSx analysis for these patients and shows promise as a cerebral dysregulation index for ECMO patients.
